# Specificity and Redundancy of Profilin 1 and 2 Function in Brain Development and Neuronal Structure

**DOI:** 10.3390/cells10092310

**Published:** 2021-09-03

**Authors:** Marina Di Domenico, Melanie Jokwitz, Walter Witke, Pietro Pilo Boyl

**Affiliations:** Institute of Genetics, University of Bonn, 53115 Bonn, Germany; marinadidomenico@gmail.com (M.D.D.); mjokwitz@uni-bonn.de (M.J.); w.witke@uni-bonn.de (W.W.)

**Keywords:** profilin, actin, cell division, neuronal precursor cells, brain development, neuronal branching

## Abstract

Profilin functions have been discussed in numerous cellular processes, including actin polymerization. One puzzling aspect is the concomitant expression of more than one profilin isoform in most tissues. In neuronal precursors and in neurons, *profilin 1* and *profilin 2* are co-expressed, but their specific and redundant functions in brain morphogenesis are still unclear. Using a conditional knockout mouse model to inactivate both *profilins* in the developing CNS, we found that threshold levels of profilin are necessary for the maintenance of the neuronal stem-cell compartment and the generation of the differentiated neurons, irrespective of the specific isoform. During embryonic development, profilin 1 is more abundant than profilin 2; consequently, modulation of profilin 1 levels resulted in a more severe phenotype than depletion of profilin 2. Interestingly, the relevance of the isoforms was reversed in the postnatal brain. Morphology of mature neurons showed a stronger dependence on profilin 2, since this is the predominant isoform in neurons. Our data highlight redundant functions of profilins in neuronal precursor expansion and differentiation, as well as in the maintenance of pyramidal neuron dendritic arborization. The specific profilin isoform is less relevant; however, a threshold profilin level is essential. We propose that the common activity of profilin 1 and profilin 2 in actin dynamics is responsible for the observed compensatory effects.

## 1. Introduction

Profilin is a monomeric (G-)actin-binding protein needed in all non-muscle cells to maintain a G-actin pool necessary for the fast actin dynamics of these cells. Compared to other G-actin-binding proteins, profilin has certain unique functions that have raised interest since its discovery more than 40 years ago [1]. Profilin has a catalytic activity that increases the exchange rate of ADP with ATP on G-actin by 1000 times [2], a function that is fundamental to generate ATP-loaded G-actin subunits that facilitate actin filament growth (F-actin) through efficient addition of the actin monomer to the filaments’ barbed end [3,4]. In addition, profilin interacts with phosphatidylinositol 4,5-bisphosphate at the plasma membrane [5], possibly to transport polymerization-competent actin monomers to the site of filament growth. Profilin’s amino-acid sequence is not particularly well conserved during evolution, but its structure and actin-binding properties, as well as the poly-l-proline (PLP)-interacting domain, are preserved across the animal kingdom and down to unicellular organisms. Already in *D. discoideum*, profilin has diverged into multiple isoforms that, in higher organisms, have acquired cell-specific expression patterns and possibly unique functions. In mammals, four profilin isoforms have been found. Profilin 1 is ubiquitously expressed [6], while profilin 2 is highly expressed in neurons in the entire central and peripheral nervous system [6,7], in testis [8], and to a much lesser extent in kidney, thymus, and spleen [6,9]. Profilin 3 and 4 are only found in testis [10,11]. Therefore, in the mammalian brain, the profilin paralogs 1 and 2 were found expressed at high levels, while they were shown to participate in different complexes and cellular pathways, most likely through a unique poly-l-proline-binding domain [6,12].

Mouse models lacking one or the other isoform have shed some light on the specific functions of profilin 1 and 2 in vivo. Conventional knockout of *profilin 1* (*Pfn1*) has shown its requirement for cell division in early embryos, with the mutant zygote not reaching the four-cell stage [9]. The absence of profilin 2 expression at early embryonic stages precludes any compensatory function. The conventional knockout of *profilin 2* (*Pfn2*), instead, resulted in a fairly normal embryonic development. Despite profilin 2 expression as early as embryonic day (E) 10.5 [9], the central nervous system (CNS) developed normally in mutant mice. However, we observed striking alterations of the synaptic physiology in mutant neurons, such as hyperexcitability of glutamatergic neurons due to increased neurotransmitter release probability, resulting in behavioral alterations and epileptic-like seizures in the animals [7]. In this mouse model, profilin 1, although present in the same neuronal compartments [13], was not able to compensate for the loss of profilin 2, suggesting that the regulation of synaptic activity in higher eukaryotes is a specific function of profilin 2. On the contrary, postnatal deletion of *profilin 1* in mature neurons of the forebrain, using a *Camk2a*-Cre mouse line, had no effect on excitatory neurons morphology and physiology [14]. Instead, deletion of *Pfn1* in neuronal precursor cells (NPCs) of the developing CNS, using a *Nes*-Cre mouse line, affected mainly radial migration of cerebellar granule neurons due to deficits in cell–cell adhesion to the radial glia cells [15,16] and reduced Purkinje cell survival starting already 2 weeks after birth in a non-cell-autonomous fashion [17]. Yet, cell proliferation and granule neuron–Purkinje cell physiology in the postnatal cerebellum were not affected. In the same context, nevertheless, defective establishment of the cleavage plane of apical radial glia cells in the ventricular zone of the telencephalon was reported. This did not appear to affect cell division, but mildly altered cortical development [18]. It is possible that, in these cell types, profilin 2 can compensate for the functions of profilin 1 in cell division that were reported for preimplantation embryonic development. 

In order to better understand the specific and redundant functions of profilin 1 and 2 during brain development and in the adult brain, we generated and analyzed two mouse models. To study brain development, we depleted both profilins at mid-gestation by combining the conventional *Pfn2* knockout allele [7] with the conditional inactivation of *Pfn1*, using the *Pfn1*-flox allele [19] and *Nes*-Cre-mediated deletion. *Nes*-Cre expression was detected in neuronal precursor cells of the CNS starting at about E9 [20]. To study the profilin requirement in adult neurons, the *Pfn1* allele was inactivated postnatally using a *Camk2a*-Cre mouse line in the mentioned *Pfn2* knockout background. *Camk2a*-Cre is expressed in forebrain glutamatergic neurons and striatal medium spiny neurons starting around postnatal day (P) 18 [21]. Our data show that embryos lacking both profilins starting from E9 develop a normal body axis, but completely lack forebrain, midbrain, and hindbrain structures, resulting in an embryonic lethal phenotype. This phenotype is completely rescued by the presence of a single *Pfn1* allele; however, it is only partially rescued by a single *Pfn2* allele. We observed a specific requirement of profilin 1 for proper embryonic cerebral cortex formation, while profilin 2 can support only hind- and midbrain development due to its lower expression level. Inactivation of both profilins in the adult brain resulted in a progressive collapse of dendritic arborizations of cortical and hippocampal pyramidal neurons. A partial rescue of the phenotype by either a single *Pfn2* or *Pfn1* allele could be observed, dependent on the expression level of the respective isoform. This finding suggests similar functions of both profilins in the adult neurons with respect to their structural integrity.

## 2. Materials and Methods

Mice. The *profilin 2* knockout (*Pfn2^tm1(lacZ)Wit^*, herein denoted as *Pfn2*^het^ and *Pfn2*^ko^) mouse model was previously described [7] (http://www.informatics.jax.org/allele/key/54529, accessed on 1 September 2021). The *profilin 1* flox mouse line (*Pfn1^tm1.1(loxPs)Ref^*), a generous gift from R. Fässler [19] (http://www.informatics.jax.org/allele/key/67261, accessed on 1 September 2021), was crossed with either the *Nes*-Cre driver line [20] (http://www.informatics.jax.org/allele/key/6206, accessed on 1 September 2021) to generate a mid-embryonic conditional deletion of *profilin 1* in neuroepithelial cells (herein denoted as n-*Pfn1*^het^ or n-*Pfn1*^ko^) or the *Camk2a*-Cre driver line [21] (http://www.informatics.jax.org/allele/key/6312, accessed on 1 September 2021) for conditional deletion of *profilin 1* in postnatal forebrain neurons (herein denoted as n-*Pfn1*^het^ or n-*Pfn1*^ko^). The *Pfn2* knockout and the *Pfn1* conditional knockout lines were crossed to generate the double *profilin* knockout (herein denoted as n-dko). All genotypes used in the experiments were obtained as littermates according to the mating scheme in Figure S1A,B. As also indicated in Figure S1, controls (ctrl) in the embryonic studies were *Pfn1^wt/wt^*;*Pfn2^wt/wt^* or *Pfn1^flx/wt^*;*Pfn2^wt/wt^*, while controls in the adult studies were *Pfn1^flx/wt^*;*Pfn2^wt/wt^* or *Pfn1^flx/flx^*;*Pfn2^wt/wt^*. Since the two Cre mouse lines were obtained by transgenic insertion, the presence of one Cre allele is indicated by the simple line name in the genotype (see Figure S1A,B). All mouse lines were extensively back-crossed (>10 times) into C57Bl/6NCrl background. Mouse genotyping was performed by PCR as schematically shown in Figure S1C. Mice were socially housed with a standard 12 h light/dark cycle at 22 °C and 50–55% humidity, with free access to water and food pellets. Line breeding and experiments were performed according to European regulations and local permission (AZ 84-02.04.2013.A233, AZ 84-02.04.2017.A088).

Histology. E11.5, E14.5, and E16.5 embryos were dissected, photographed, and fixed O/N in 4% formaldehyde solution in PBS at 4 °C, then dehydrated in an increasing alcohol concentration series followed by xylene, then impregnated in paraffin O/N at 60 °C, and lastly transferred into plastic molds to solidify according to a classical protocol. E14.5 and E16.5 embryos were decapitated to facilitate paraffin embedding. The embryos were then serially cut sagittally using a microtome with 8 μm thickness. The sections were then rehydrated with an inverse xylene/alcohol series and stained with Meyer’s hemalum (Merck KGaA, Darmstadt, DE, 109249) and eosin Y (Sigma, St. Louis, MO, USA, HT110132).

Immunofluorescence. E11.5 embryos were fixed O/N in 4% formaldehyde at 4 °C and exchanged in sucrose (15% and 30% in PBS), then frozen in Tissue-Tek^®^ in a mold on powdered dry ice, and sagittally cut with 14 μm thickness at a cryostat. Sections were collected on Superfrost slides, dried for 30 min at RT and stored at −80 °C. For the immunofluorescence, the frozen slides were quickly dipped in 4% formaldehyde to postfix the slices and blocked for 2 h at RT in 5% goat serum and 2% DMSO in TBS-T (Tris-buffered saline with 0.05% TritonX-100). Primary antibodies were diluted in blocking solution and incubated O/N in a humidified chamber. Washes were performed with TBS-T, and secondary antibody incubation was again conducted in blocking solution for 2 h at RT. Primary antibodies used were anti-phospho-histone 3 (Ser10) rabbit pcl (Upstate/Merck, 06-570, 1:500) and anti-βIII-tubulin mouse mcl (Promega, Madison, WI, USA, G7121, 1:1000). Alexa 488-conjugated anti-rabbit and Alexa 594-conjugated anti-mouse secondary antibodies (Thermo Fisher Scientific, Waltham, MA, USA, 1:400) were used. Nuclei were stained with DAPI (Sigma, 0.2 μg/mL). Images were taken with a Keyence (Osaka, Japan) BZ-9000 microscope.

P80–90 mice were sacrificed by cervical dislocation, and the brain was quickly dissected on ice, washed in PBS, and fixed in 4% formaldehyde in PBS for 40 h at 4 °C. The brain was then sliced coronally using a vibratome with 40 μm thickness. Slices were stored in PBS at 4 °C until use. Immunofluorescence staining was performed according to the classical protocol for floating sections. In brief, sections were treated with 50 mM NH_4_Cl for 1 h at RT, then blocked in 5% NGS and 2% DMSO in TBS-T O/N at 4 °C, incubated in primary antibodies diluted in blocking solution O/N at 4 °C, washed in TBS-T, incubated in secondary antibodies diluted in blocking solution for 2 h at RT, washed again, and mounted in 20% Mowiol with 5% *N*-propylgallate. Primary antibodies used were anti-neurogranin rabbit pcl (Proteintech, Rosemont, IL, USA, 10440-1-AP, 1:500), followed by Alexa 488-conjugated anti-rabbit secondary antibodies. Draq5 (Abcam, Cambridge, UK, 1:1000) was used for nuclear labeling in the red spectrum. Imaging was performed with a Zeiss LSM510 confocal microscope.

Golgi staining. The FD Rapid GolgiStain™ Kit (FD NeuroTechnologies, Columbia, MD, USA) was used, according to the manufacturer’s instructions. Briefly, P80–90 mice were sacrificed by cervical dislocation, and the brain was quickly dissected on ice, washed in PBS, and impregnated in staining solution for 15 days. It was then washed in the provided solution for 3 days, cut coronally with 250 μm thickness, applied to chrom-alum gelatin-coated slides, dried O/N, developed, dehydrated, and mounted with Entellan (Merck). Imaging was performed with a Keyence BZ-9000 microscope taking z-stacks of entire V layer neurons in the motor cortex region. The branching analysis was performed manually and blind of the analyzed genotype, as much as possible given the strong phenotype in some of the genotypes, counting the branches and their length using Image J. The full projection image used for the analysis was always compared with the original stack to avoid false attribution of crossing branches from neighboring stained neurons.

Western blotting. After dissection, embryonic and adult brain tissue was quickly frozen in N_2(l)_ and stored at −80 °C until lysed. Total tissue lysis was performed in adequate volumes of 2× Laemmli buffer in a glass Teflon tissue homogenizer with an electrically controlled rotation at 600 rpm, to obtain a protein concentration between 2 and 4 mg/mL. The concentration was estimated with the Bradford method using BSA to produce a standard curve. Western blotting was performed according to standard protocols, loading 2.5 to 5 μg of protein for more accurate relative quantification. Submerged blotting was adapted when necessary to the small-molecular-weight proteins, transferring at 160 mA for 30 min. Antibody incubation and washes were performed in TBS-T (0.05% Tween-20) at pH 8.2. Primary antibodies used were homemade anti-PFN2 3003 (1:500) and anti-PFN1 T1 (1:1000) rabbit sera [6], anti-cofilin 1 KG60 (1:500) rabbit serum [22], anti-phospho-cofilin 1 (Ser 3) rabbit pcl (Cell Signaling Technology, Danvers, MA, USA, 3313, 1:500), anti-actin mouse mcl (MP Biomedicals, Santa Ana, CA, USA, 08-691002, clone C4, 1:5000), anti phospho-H3 (Ser 10) rabbit pcl (Upstate/Merck 06-570, 1:1000), anti-γ-tubulin mouse mcl (Sigma T6557, 1:2000), anti-phospho-CDC2/CDK1 (Tyr15) rabbit pcl (Cell Signaling Technology 9911, 1:1000), anti-PTTG1 rabbit pcl (Abcam ab26273, 1:1000), anti-vimentin rabbit pcl (Abcam ab92547, 1:500), and anti-GFAP rabbit pcl (Sigma G9269, 1:1000). Chemiluminescence images were acquired with a LAS4000-Mini Imager (FujiFilm/Cytiva, Marlborough, MA, USA). Coomassie staining of the membrane was performed after the antibody detection following a standard protocol. Briefly, the membrane was incubated in 0.2% Coomassie R250 in 50% methanol/10% acetic acid for 10–15 min, then washed 2× in 40% methanol/10% acetic acid for 10 min, and lastly left in 10% methanol/10% acetic acid for 1 h, air-dried, and imaged.

F/G-actin ratio. Dissected cortices from P120 mice were lysed on ice in McRobbie’s PHEM buffer (PIPES-Na 60 mM, HEPES-Na 25 mM, EGTA 10 mM, MgCl_2_ 2 mM at pH 6.9) with 1% TritonX-100 in a glass-Teflon homogenizer at a constant electrically regulated speed of 600 rpm. Lysates were spun at 10,000× *g* for 10 min at 4 °C, the supernatant was set aside, and the pellet was dried of any supernatant, before fragmenting and resuspending in an equal volume of PHEM buffer with 1% TritonX-100. Both fractions were then complemented with 1× Laemmli buffer and boiled at 100 °C for 10 min. Equal volumes of the pellet (F-actin) and supernatant (G-actin) fractions were loaded on a gel and quantified by Western blotting to calculate the F/G-actin ratio.

Statistics. Statistical evaluation was performed with Microsoft Office Excel, GraphPad, or Minitab software. Applied tests are specified in the figure legends. In general, one-way ANOVA was applied when comparing the four genotypes, followed by Dunnett’s post hoc test to compare the mutant genotypes to the control. Two-sided Welch’s *t*-test (not assuming equal variance of the samples) was used when comparing the *profilin* double knockouts to controls, since there is no guarantee that, in the mutant mice, the variance was the same as in the controls. The one-sided *t*-test was applied only on occasions where, due to the genetic manipulation, it was not possible to have an overlap of the mutant and the control data distributions, mainly to evaluate the significance of the profilin and the actin loss in the profilin double mutants.

## 3. Results

**Figure 1:**
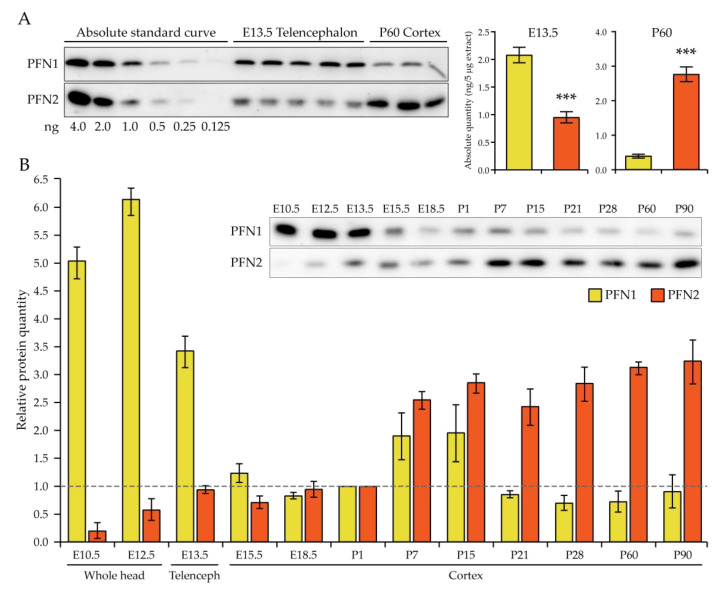
Profilin 1 and 2 expression in the neural tissue. (**A**) Western blots and relative histograms representing the absolute quantification of profilin 1 and profilin 2 in total protein extracts from E13.5 telencephali and P60 cortices from C57Bl/6NCrl mice, using 4, 2, 1, 0.5, 0.25, and 0.125 ng of the respective recombinant profilin for the standard curve. To avoid membrane or ECL signal saturation artefacts, only 2.5 μg of protein extract was loaded onto the polyacrylamide gel, except for P60 cortices in the PFN1 blot, where 5 μg was used due to the low PFN1 expression. Telencephalic and cortical extracts were calibrated with Coomassie staining of the membrane. At E13.5, profilin 1 is double the level of profilin 2 (Welch’s double-sided *t*-test, *p* < 0.001), whereas, in P60 cortices, profilin 2 is sevenfold the level of profilin 1 (Welch’s double-sided *t*-test, *p* < 0.001); *n* = 5 E13.5 wt telencephali, *n* = 3 wt P60 cortices. (**B**) Relative quantification histogram of profilin 1 and 2 in protein extracts of E10.5 and 12.5 whole embryo heads, E13.5 telencephali, and E15.5, E18.5, P1, P7, P14, P21, P28, and P60 cortices of C57Bl/6NCrl mice. In the inset, sample blots for each profilin are shown. The expression pattern follows opposite trends. Profilin 1 is higher expressed during early nervous system development, rapidly decreasing already at E15.5 and ending at minimal levels in adulthood, after an increase in the temporal window of synaptogenesis; profilin 2, on the other hand, is expressed at its lowest levels during early embryonic brain development and reaches a first plateau at E13.5, before rising to maximal expression at the start of synaptogenesis (P7), remaining constantly high throughout adulthood; *n* = 3 wt tissues at each time point. Error bars represent the SEM; *** *p* ≤ 0.001.

Previous work has shown the relative expression of profilin 1 and profilin 2 during embryonic development in protein extracts of the entire embryo [9] or from E11.5 to E16.5 entire embryo heads [23]. In these extracts, the neural tissue is diluted to a different extent by the non-neural tissue, thus masking the specific expression of profilins in the developing nervous system. This is particularly relevant for profilin 2, since this isoform is well expressed in the neurons but virtually not expressed in the surrounding non-neuronal tissue [6,9,23], resulting in an apparent very low expression during embryonic development. In contrast, profilin 1 is ubiquitous, thus appearing to be highly expressed at fairly constant levels at all embryonic stages. In order to obtain a faithful picture of profilin 1 and 2 expression levels in the developing neural tissue, two approaches were employed. First, an absolute quantification of profilin 1 and profilin 2 in E13.5 telencephalic protein extracts of C57Bl/6NCrl mice was performed, comparing to serial dilutions of the recombinant profilins (Figure 1A). Second, the relative expression of profilins in the neural tissue was determined from early embryonic brain development to adult cortex in C57Bl/6NCrl mice, starting with E10.5 and E12.5 embryo head extracts, then E13.5 telencephalic extracts, and lastly E15.5, E18.5, P1, P7, P15, P21, P28, P60, and P90 cortical extracts (Figure 1B).

In E13.5 telencephalon, profilin 2 was expressed at a similar level to profilin 1, being only half of the latter (Figure 1A). Instead in the cortex at P60, profilin 2 expression exceeded profilin 1 by about sevenfold, showing an opposite expression pattern of the two profilins during development. Profilin 1 expression started high in the early brain development stages until E13.5 but was significantly decreased already by E15.5, remaining stable until P1. Postnatally, a slight surge in profilin 1 expression was seen again between P7 and P15, at the apex of spinogenesis, with a final drop by P21, before then stabilizing at constant levels throughout adulthood, about fivefold lower than in E13.5 telencephalon (Figure 1B). Profilin 2, in contrast, was barely expressed at early brain development stages, but steadily increased its expression until E13.5, remained stable until birth, and then rose sharply at P7, when spinogenesis starts in the mouse cortex. Then, the expression level remained stable into adulthood, about threefold higher than in E13.5 telencephalon.

### 3.1. Profilin Roles during Embryonic Brain Development

Having established that profilin 1 and 2 expression levels are only marginally different in the early neural tissue, we addressed the question of whether their functions were redundant or if specific functions could be dissected during embryonic CNS development. We used a double *profilin* knockout mouse model, where the floxed *profilin 1* gene was inactivated by a *Nes*-Cre driver in a *profilin 2* knockout background. In addition to the double mutants, we included in the analysis the single *profilin* allele genotypes. For simplicity, the genotypes of the embryos and the mice are termed ctrl, n-dko (neural *profilin 1* knockout and *profilin 2* knockout), n-*Pfn1*^het^;*Pfn2*^ko^ (the single *profilin* 1 allele), and n-*Pfn1*^ko^;*Pfn2*^het^ (the single *profilin 2* allele), where “n” indicates the conditional inactivation of *Pfn1* in the neural tissue. Interestingly, despite all genotypes being found according to the expected Mendelian ratio up to E16.5, no n-*Pfn1*^ko^;*Pfn2*^het^ and n-dko animals, both lacking *Pfn1*, were found viable at birth, indicating that a minimal profilin amount is needed during early CNS development and for viability, which is provided by a single *Pfn1* allele. On the contrary, a single *Pfn2* allele is not sufficient for survival. In order to study the phenotypic cause of embryonic lethality, we first evaluated the levels of profilin 1 and profilin 2 in the head of the mutant embryos at E11.5, since the *Nes*-Cre transgene expression is reported to start around E9 [20]. Embryos heterozygous for *Pfn1* in the neural tissue showed already a strong reduction in protein expression, down to 35% of the control levels, while embryos that were knockout for *Pfn1* in the neural tissue had a further reduction to 15–20% of control levels (Figure S2A,B), irrespective of whether they retained one *Pfn2* allele or none. The residual profilin 1 was presumably the contribution of the non-neural tissue in the E11.5 head extracts. Embryos that were heterozygous for *Pfn2* expressed about 40% of PFN2 compared to control levels, while homozygous embryos for *Pfn2* had no protein expression, as expected in a conventional knockout model (Figure S2A,C). Interestingly, the loss of profilin 1 was accompanied by a 40% decrease in total actin levels in E11.5 embryo heads, irrespective of the presence of a *profilin 2* allele. An intermediate loss of actin was seen in embryos retaining a single *profilin 1* allele and lacking both *profilin 2* alleles (Figure S1D). We hypothesized that a loss of actin and, consequently, actin polymerization in the n-dko embryos would affect actin dynamics in general. As a known parameter for actin dynamics, we measured the levels of cofilin 1 phosphorylation on serine 3, which correlates with reduced F-actin depolymerizing activity. We found that S3 phosphorylation was greatly increased in *profilin* n-dko embryos, as well as partially in n-*Pfn1*^ko^;*Pfn2*^het^ embryos (Figure S2E,F), without a significant change in the expression level of cofilin 1. Together, our data show that actin dynamics are severely impaired when profilins are depleted.

**Figure 2:**
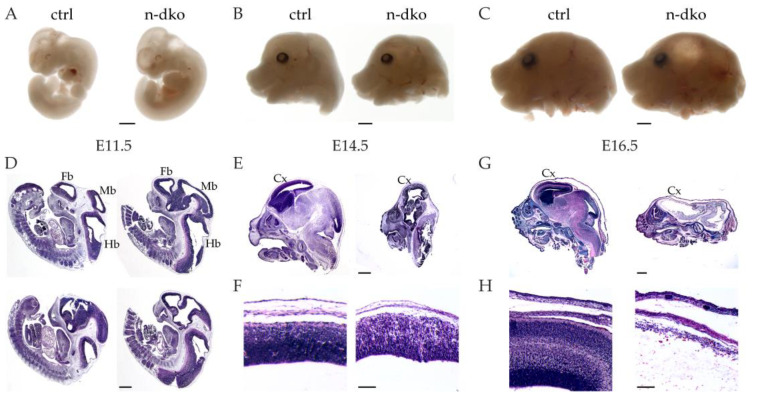
Histological analysis of neural profilin 1 and 2 knockout (n-dko) embryos. (**A**) E11.5 n-dko dissected embryos show no evident anatomical differences from control littermates. (**B**) E14.5 n-dko embryos have a smaller head than controls. (**C**) In E16.5 n-dko embryos, a translucent bubble in place of the cerebral cortex region is visible. (**D**) Hemalum/eosin (H&E)-stained sagittal sections of E11.5 n-dko embryos show no major defects in the patterning of the brain except an expansion of the hindbrain vesicle, possibly due to a delay in neural tube closure in the cervical region. (**E**) H&E-stained sagittal sections of E14.5 n-dko embryos show severe developmental defects in the forebrain, midbrain, and hindbrain. (**F**) The developing cortex of E14.5 n-dko embryos is severely compromised and does not show any layering, while cell density is strongly reduced. (**G**) In H&E-stained sagittal sections of E16.5 n-dko embryo heads, no brain structure can be identified, and the region is virtually empty. (**H**) Magnification of the cortical area shows the complete absence of the cortex in E16.5 n-dko embryos. Fb: forebrain, Mb: midbrain, Hb: hindbrain, Cx: cortex. Scale bars: 2 mm in (**A**–**C**); 1 mm in (**D**,**E**,**G**); 200 μm in (**F**,**H**).

To study the effect of reduced actin dynamics on brain development, we performed histological analysis of the double profilin mutants at E11.5, E14.5, and E16.5. N-dko embryos at E11.5 did not show significant differences in the size and body axis compared to controls (Figure 2A). Starting at E14.5, however, the head of the n-dko embryos appeared smaller compared to control littermates (Figure 2B), and, at E16.5, a clear translucent region was evident in place of the developing cerebral cortex (Figure 2C).

No major patterning alterations were detected in sagittal sections of E11.5 embryos in the brain, with the forebrain, midbrain, and hindbrain vesicles clearly visible. Consistently, we observed an expansion of the hindbrain vesicle in n-dko embryos, possibly due to a delay in neural tube closure in the cervical region (Figure 2D). At E14.5, a major impairment of brain development was evident (Figure 2E), with all ventricles enlarged and a decreased cell number in the CNS territories, clearly visible in the developing cortex that showed decreased cell density (Figure 2F). At E16.5, there was no brain structure visible in n-dko embryos and the brain region appeared virtually empty (Figure 2G), explaining the translucency observed in the embryo head at dissection. Any cortical structure was completely missing, and only a thin cell layer remained in its place (Figure 2H).

**Figure 3:**
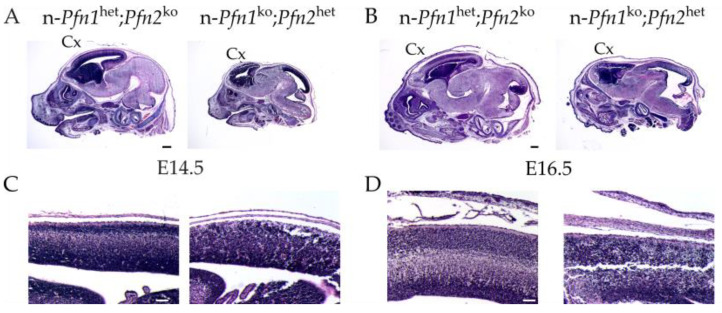
Histological analysis of single profilin 1 and single profilin 2 allele embryos. (**A**) A sample H&E-stained sagittal section of single *Pfn1* allele (n-*Pfn1*^het^;*Pfn2*^ko^) embryo head at E14.5 shows no developmental defects, while a sample section from single *Pfn2* allele (n-*Pfn1*^ko^;*Pfn2*^het^) embryo head shows reduced size, similarly to the n-dko genotype, and less compact tissue, even though development does not appear completely impaired. (**B**) Sample H&E-stained sagittal section of single *Pfn1* allele E16.5 embryo head shows normal development, while the single *Pfn2* allele embryos appear strongly affected, with a collapsing forebrain and reduced mid-/hindbrain size. (**C**) Cortical layering at E14.5 is not affected in n-*Pfn1*^het^;*Pfn2*^ko^ embryos, while it is completely disrupted in n-*Pfn1*^ko^;*Pfn2*^het^ embryos, similarly to n-dko embryos (see Figure 2F). (**D**) The cortex at E16.5 shows perfect layering in single *Pfn1* allele embryos, but is disordered in single *Pfn2* allele embryos. Scale bars: 1 mm in (**A**,**B**); 200 μm in (**C**,**D**).

The dramatic developmental phenotype of the double *profilin* knockout prompted us to study the rescue capacity of profilin 1 and profilin 2 by analyzing two additional mutant genotypes carrying a single *profilin 1* allele (n-*Pfn1*^het^;*Pfn2*^ko^) and a single *profilin 2* allele (n-*Pfn1*^ko^;*Pfn2*^het^). For this analysis, we focused at E14.5 and E16.5, the time points where the phenotype of the double mutant was most pronounced. Interestingly, the two genotypes showed a strikingly different morphology. The n-*Pfn1*^het^;*Pfn2*^ko^ animals, with a single *Pfn1* allele, were similar to the controls at E14.5, as well as E16.5 (Figure 3A,B left side, and see Figure 2E–G), while the n-*Pfn1*^ko^;*Pfn2*^het^ animals, with a single *Pfn2* allele, appeared to be developmentally impaired already at E14.5, with a smaller head size. The developmental delay was even more striking at E16.5, when void areas started to appear, similarly to the n-dko embryos, although with a time delay (Figure 3A,B right side, and see Figure 2E,G). At this juncture, it is noteworthy to mention that n-*Pfn1*^ko^;*Pfn2*^het^ mice are embryonic lethal, like the n-dko mice. The histological analysis of the cortex of n-*Pfn1*^ko^;*Pfn2*^het^ embryos confirmed an altered structure and cell density already at E14.5 with complete absence of layering and loss of compactness at E16.5 (Figure 3C,D). On the contrary, cortical development at E14.5 and E16.5 appeared normal in n-*Pfn1*^het^;*Pfn2*^ko^ embryos, and mutant mice were born and viable.

**Figure 4:**
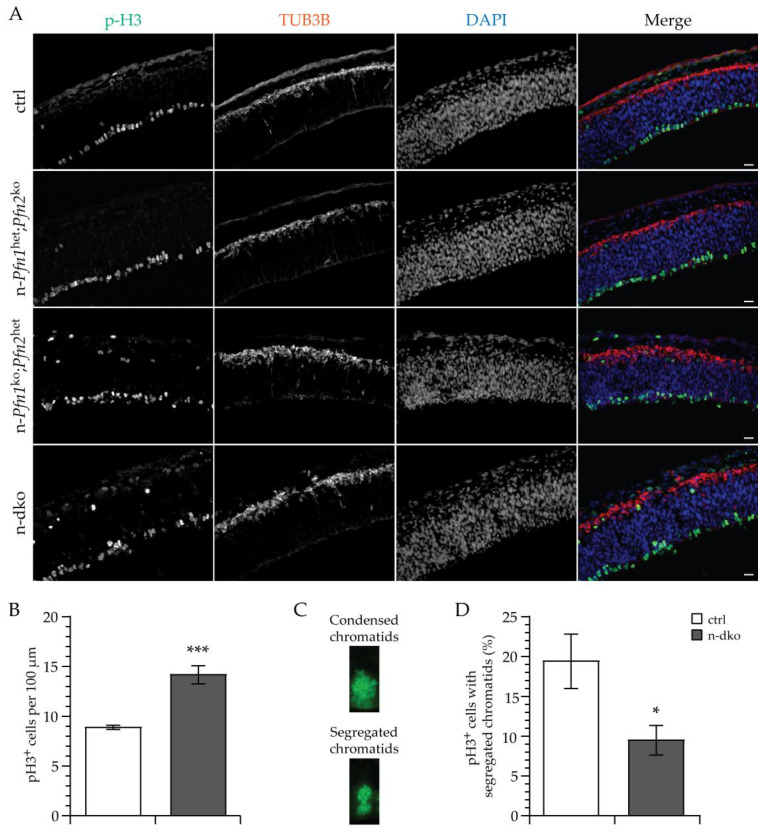
Mitotic neural precursor cells (NPCs) in the VZ are disarrayed and increased in E11.5 n-dko embryos. (**A**) Immunofluorescence staining of E11.5 cryosections with anti-p-H3 (S10 phospho-histone 3) antibodies, green, to label dividing NPCs, anti-TUB3B (β3-tubulin) antibody, red, to label differentiated neurons on the pial surface, and DAPI, blue, to label cell nuclei, shows a disarrayed layer of dividing NPCs and ectopic p-H3 labeling in the forebrain region of n-dko, as well as n-*Pfn1*^ko^;*Pfn2*^het^ embryos. On the other hand, embryos carrying a single *Pfn1* allele, n-*Pfn1*^het^;*Pfn2*^ko^, appear unaffected. Scale bar: 20 μm. (**B**) Quantification of p-H3 positive cells in the VZ of E11.5 embryos shows increased linear density in n-dko *profilin* mutants (14.17 ± 0.89/100 μm) compared to controls (8.91 ± 0.20/100 μm, two-sided Welch’s *t*-test *p* < 0.001, *n* = 8/2 sections/embryos for ctrl and *n* = 11/2 for n-dko). (**C**) Examples of p-H3-labeled condensed and segregated chromatids in NPCs, with the latter indicating the progression of cell division to the telophase stage. (**D**) Quantification of p-H3^+^ NPCs with segregated chromatids shows a significant 50% decrease in n-dko embryos (9.5% ± 1.87% compared to 19.42% ± 3.4% in controls, two-sided Welch’s *t*-test, *p* = 0.0215, *n* = 8/2 sections/embryos for ctrl and *n* = 11/2 for n-dko); * *p* ≤ 0.05, *** *p* ≤ 0.001. Scale bar: 20 μm.

In order to obtain further insight into the cellular mechanisms via which alterations in actin dynamics can affect the developmental program of the brain, we focused on the onset of profilin loss at E11.5 and studied cell division in the ventricular zone (VZ), where expansion of the stem-cell pool and birth of the cortical neurons occur. Symmetric and asymmetric cell division of neural precursor cells (NPCs) in the ventricular zone of all three brain vesicles is the primary source of cells that, through radial migration, will populate the growing brain, but this is particularly important for the forebrain vesicle. We, therefore, labeled mitotic cells in the forebrain with antibodies directed against histone 3 phosphorylated on Ser10 (p-H3) [24] and marked differentiated neurons with anti-β3-tubulin (TUB3B) antibodies [25] in embryonic sections of all four described genotypes. We found that the dividing cells in the ventricular zone were disarrayed and misplaced in n-dko embryos and similarly in n-*Pfn1*^ko^;*Pfn2*^het^ embryos. On the contrary, n-*Pfn1*^het^;*Pfn2*^ko^ embryos with a single *profilin 1* allele were rescued and almost normal compared to control littermates (Figure 4A). A very similar phenotype was seen in the midbrain (Figure S3A). Since the p-H3-labeled layer in n-dko embryos appeared thicker, we calculated the linear density of dividing NPCs in control and double profilin mutant embryos. The linear density of p-H3-positive cells was almost double in the forebrain of n-dko embryos (Figure 4B), as well as in their midbrain (Figure S3B), compared to controls. At first glance, the increased number of p-H3-positive cells could seem contradictory with the established requirement of profilin in cell division and with the phenotype observed at later stages of brain development (see Figure 2). We reasoned that the increased number of cells with condensed metaphase chromosomes in fact reflected a cell-cycle arrest in NPCs depleted of both profilins. To confirm our hypothesis, we compared the percentage of dividing NPCs displaying segregated chromatids, thus exiting cell division, rather than condensed ones (Figure 4C and Figure S3C), in profilin n-dko and control embryos. Indeed, the percentage of cells with segregated chromatids in n-dko embryos was half that in controls in the forebrain (Figure 4D), as well as in the midbrain (Figure S3D).

**Figure 5:**
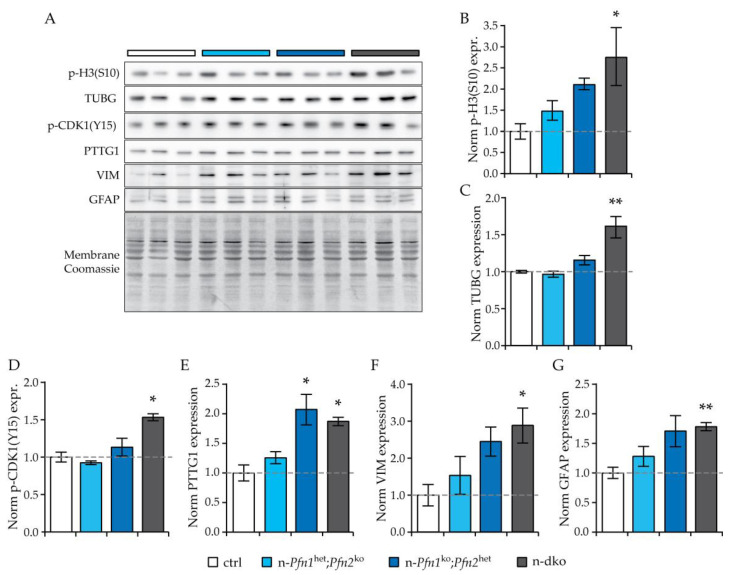
Markers of cell division G2 phase and of neural precursor cells are increased in n-dko embryos. (**A**) Western blots of the markers tested on E11.5 embryo head extracts of profilin mutants and controls. Total lane protein quantified by Coomassie staining of the membrane was used for calibration (a sample membrane is shown).Three extracts per genotype were analyzed, as indicated by the color bars. Histograms representing normalized quantification of (**B**) histone 3 Ser10 phosphorylation (p-H3(S10)), a marker of chromosome condensation, (**C**) γ-tubulin (TUBG), (**D**) CDK1 Tyr15 phosphorylation (p-CDK1(Y15)), and (**E**) PPTG1 (also known as securin) show increased expression in neural double *profilin* knockout (n-dko) embryos and an intermediate increase in the presence of a single *Pfn2* allele (n-*Pfn1*^ko^;*Pfn2*^het^). The findings point to an arrest of NPCs cell division in the G2-phase. Histograms representing normalized quantification of the intermediate filament proteins (**F**) vimentin (VIM) and (**G**) glial fibrillary acidic protein (GFAP) show higher expression in n-dko embryos and an intermediate increase in n-*Pfn1*^ko^;*Pfn2*^het^ embryos, suggesting an accumulation of cells with NPC identity. For each marker, one-way ANOVA followed by Dunnett’s post hoc test was applied, to compare to ctrl levels; *n* = 3 per genotype; * *p* ≤ 0.05, ** *p* ≤ 0.01.

Since the cell-cycle arrest appeared to affect all brain vesicles, we prepared E11.5 embryo head protein extracts and studied the phenotype at the biochemical level including a wider selection of cell division markers and extending the analysis to the single *profilin* allele genotypes in order to further dissect the specific functions of the two profilins in brain development. Western blotting confirmed a 2.5-fold increase in p-H3 levels in n-dko E11.5 embryos compared to controls, as well as an intermediate increase in n-*Pfn1*^ko^;*Pfn2*^het^ embryos, missing profilin 1, while no increase in p-H3 was detected in n-*Pfn1*^het^;*Pfn2*^ko^ embryos expressing profilin 1 (Figure 5A,B). Chromosome duplication and condensation in the S-phase are accompanied by centrosome duplication [26], which can be monitored by the levels of γ-tubulin (TUBG). In agreement with the higher p-H3 levels, the amount of TUBG was also significantly increased in n-dko and n-*Pfn1*^ko^;*Pfn2*^het^ embryos (Figure 5A,C). In late G2 phase, the cyclin-dependent kinase 1 (CDK1), also known as M-phase promoting factor or CDC2, is de-repressed by dephosphorylation of threonine 14 and tyrosine 15 by the cell division cycle protein phosphatase CDC25C [27,28] to guide the transition into M-phase. We, therefore, measured Tyr15 phosphorylation of CDK1 in E11.5 head extracts of profilin mutants. Tyr15 phosphorylation was 1.5-fold higher compared to controls in n-dko embryos (Figure 5A,D), indicating an arrest or retardation of cell division at the G2/M transition phase. Another factor that is specifically regulated during cell division is the pituitary tumor-transforming gene 1 (PTTG1), also known by the name securin, which is degraded upon entry of the cells into M-phase because its function is to prevent separins (in humans, known as extra spindle polebodies like proteins, ESPLs) from promoting sister chromatid separation. Since we observed a decreased number of NPCs with segregated chromatids (Figure 4D and Figure S3D) in n-dko embryos, we evaluated the levels of PTTG1 in profilin mutants. We found that PTTG1 was significantly higher by approximately twofold in n-dko and n-*Pfn1*^ko^;*Pfn2*^het^ embryos, but was unaltered in n-*Pfn1*^het^;*Pfn2*^ko^ embryos retaining a single *profilin 1* allele (Figure 5A,E), in agreement with the previous markers and with the reduced number of dividing cells with segregated chromatids in the n-dko embryos. Lastly, reasoning that the arrest in cell division might result in an accumulation of cells retaining NPC identity, we measured the levels of two specific markers of apical radial glia cells, the intermediate filament proteins vimentin (VIM) [29] and glial fibrillary acidic protein (GFAP) [30]. Both VIM and GFAP were significantly increased in n-dko embryos and similarly in n-*Pfn1*^ko^;*Pfn2*^het^ embryos, while no significant change was observed in n-*Pfn1*^het^;*Pfn2*^ko^ embryos, retaining a single *profilin 1* allele, compared to controls (Figure 5A,F,G).

In summary, embryos retaining a single *Pfn1* allele were able to develop quite normally, while, in embryos with only one *Pfn2* allele or no *profilin* at all, the cell division of NPCs was defective, producing severe brain development deficits and perinatal lethality.

### 3.2. Profilins’ Roles in the Adult Brain

In the previous section, we established that profilin 1 has a major role during embryonic brain development that can be only minimally compensated for by profilin 2. The next question we asked concerned the specific roles of profilin 1 and 2 in the adult brain, once brain morphogenesis is complete. To answer this question, we capitalized on a postnatal deletion mouse model of *profilin 1* in a *profilin 2* knockout background. This would provide us with normally developed brains at the beginning of our studies. We obtained the conditional deletion of *profilin 1* through a *Camk2a*-Cre transgenic allele, whose expression starts around postnatal day (P) 19, i.e., close to the end of the spinogenesis period, in hippocampal and cortical glutamatergic neurons [21]. We verified that an efficient deletion of profilin 1 in cortex and hippocampus occurred already at P24. We, therefore, decided to study the effect of postnatal deletion of *profilin 1* and *2* in the forebrain at P80–90, about 60 days after the glutamatergic neurons lost all profilin. In P80–90 cortex, n-dko mice expressed 30% of profilin 1 and no profilin 2 (as expected in a conventional knockout model) compared to controls, while mice with a single *profilin 1* allele (n-*Pfn1*^het^;*Pfn2*^ko^) had about 70% of profilin 1 and no profilin 2 compared to controls. Mice with a single *profilin 2* allele (n-*Pfn1*^ko^;*Pfn2*^het^) expressed 40% of profilin 1, consistently 10% more than n-dko mice, and 50% of profilin 2 (Figure S4). Profilin 1 is ubiquitously expressed, while the conditional knockout acts only on glutamatergic neurons in the cortex, which account for about 25% of the total cells [31]; therefore, it was not surprising that n-*Pfn1*^het^;*Pfn2*^ko^ still expressed 70% of profilin 1 in a cortical lysate and mice with a conditional knockout of *profilin 1* in glutamatergic neurons still expressed 30–40% of profilin 1. We also measured the absolute protein levels of profilin 1 and 2 in the cortex of P60 wt mice and determined that profilin 2 is about sevenfold more abundant than profilin 1 (Figure 1A). One should keep in mind that, in n-*Pfn1*^ko^;*Pfn2*^het^ mice, preserving a single *Pfn2* allele, the neurons contain about sevenfold more profilin than in n-*Pfn1*^het^;*Pfn2*^ko^ mice that bear a single *Pfn1* allele.

**Figure 6:**
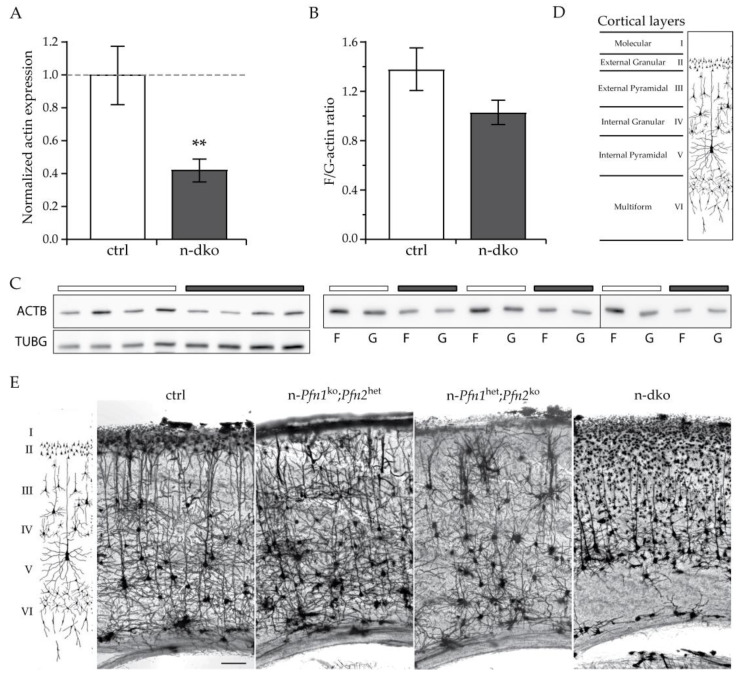
Reduction in total and filamentous actin affects neuronal morphology and survival in the cortex of n-dko mice. (**A**) Total actin (ACTB) in cortical extracts is reduced by 60% in n-dko mice compared to controls (one-sided Welch’s *t*-test, *p* = 0.0268, *n* = 4 per genotype). Actin levels were calibrated according to γ-tubulin (TUBG) expression. (**B**) The filamentous to monomeric actin ratio (F/G-actin ratio) is reduced in n-dko mice compared to controls; *n* = 3 per genotype. Error bars represent the SEM. (**C**) Western blots used for the quantifications shown in (**A**,**B**). (**D**) Schematic drawing illustrating the mouse cortical layers as they would appear in a Golgi staining. (**E**) Sample images of matched Golgi-stained cortical coronal slices in the motor cortex region of ctrl, n-*Pfn1*^ko^;*Pfn2*^het^, n-*Pfn1*^het^;*Pfn2*^ko^, and n-dko P80–90 mouse brains. The scheme on the left is the same as in (**D**) and helps in discriminating the neuronal layers in the Golgi-stained cortical columns shown on the right. A progressive loss of neuronal cell bodies and dendritic arborization can be seen as profilin levels decrease in the four panels from left to right, particularly evident for the neurons of the upper layers II and III. Scale bar: 100 µm; ** *p* ≤ 0.01.

Reasoning that removal of both profilins might affect the actin cytoskeleton, we quantified total actin and studied the ratio of filamentous (F) actin to monomeric (G) actin in cortical extracts of P80-90 n-dko mice. The total actin content was decreased by 60% in n-dko mice (Figure 6A,C), in agreement with the notion that actin must be kept in complex with a monomeric actin binding protein in order to avoid spontaneous polymerization [32]. Moreover, the steady-state F/G-actin ratio in the cortex was found mildly decreased by 25% in n-dko mice compared to controls (Figure 6B,C). The alterations in the actin levels, as well as the F/G-actin ratio reduction, suggested cytoskeletal deficits in the mutant neurons that could affect neuronal morphology. Therefore, we performed Golgi staining of P80–90 mouse brains to verify neuronal morphology in the cortex and hippocampus. The cortical structure is particularly suitable to detect changes due to its well-known stratified organization and elaborated pyramidal neuron layers (Figure 6D). In summary, we observed an increasing loss of neuronal complexity as the overall level of profilins was reduced (Figure 6E). Morphological alterations increased as the profilin levels decreased from a single *profilin 2* allele (n-*Pfn1*^ko^;*Pfn2*^het^) to a single *profilin 1* allele (n-*Pfn1*^het^;*Pfn2*^ko^) and finally to no profilin at all in n-dko mice. Both neuronal density and dendritic complexity were progressively reduced, the latter particularly in the neurons in the upper layers II and III.

**Figure 7:**
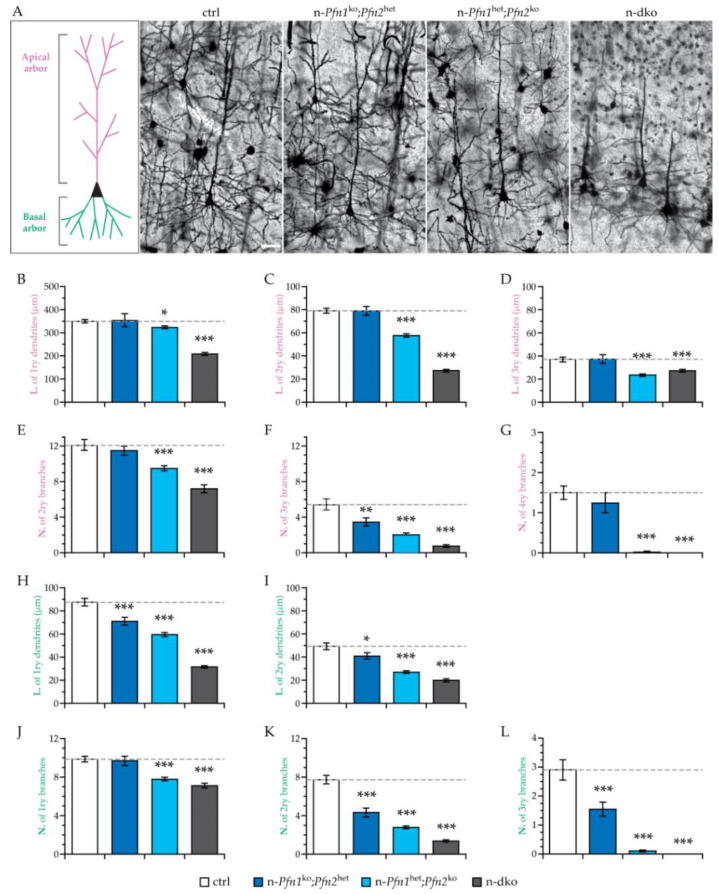
Length and complexity of dendritic arborizations in layer V cortical neurons are reduced proportionally to the profilin expressed. (**A**) Sample images of Golgi-stained layer V cortical neurons in control (ctrl), n-*Pfn1*^ko^;*Pfn2*^het^, n-*Pfn1*^het^;*Pfn2*^ko^, and n-dko P80–90 mouse brain slices. The drawing on the left side is a schematic representation of a wt layer V cortical neuron in scale with the neurons in the images. Length (L) of apical (**B**) primary dendrites, as well as of (**C**) secondary and (**D**) tertiary branches of layer V cortical neurons, is reduced compared to controls proportionally to the reduction in expressed profilin; one *Pfn2* allele is sufficient to rescue dendrite length but not one *Pfn1* allele. The number (N) of (**E**) secondary, (**F**) tertiary, and (**G**) quaternary apical branches is strongly reduced in profilin double mutant neurons compared to controls proportionally to the reduction in profilin levels. No quaternary branches were found in n-dko and single *Pfn1* allele cortical neurons. Length (L) of basal (**H**) primary and (**I**) secondary dendrites is reduced in profilin mutant neurons compared to controls, similarly to the apical dendrites. The number (N) of basal (**J**) primary, (**K**) secondary, and (**L**) tertiary branches is strongly reduced in n-dko neurons compared to controls, similarly to the apical dendrites. No tertiary branches were found in n-dko and single *Pfn1* allele cortical neurons. For each parameter, one-way ANOVA with Dunnett’s post-hoc test was applied to compare to ctrl values; *n* = 35/2 (neurons/mice) per genotype. Error bars represent the SEM; * *p* ≤ 0.05, ** *p* ≤ 0.01, *** *p* ≤ 0.001. Scale bar: 40 μm.

In order to substantiate these observations, we performed a quantitative analysis of both the apical and the basal dendritic arbors in cortical layer V pyramidal neurons, since these neurons are well characterized and isolated enough in Golgi staining. The length of apical primary dendrites, as well as the number and length of all higher-order branches, of layer V neurons, depended on profilin dosage (Figure 7A–G), irrespective of the isoform. The length of the dendrites (Figure 7B–D) was preserved in the presence of a single *profilin 2* allele (n-*Pfn1*^ko^;*Pfn2*^het^), but a single *profilin 1* allele (n-*Pfn1*^het^;*Pfn2*^ko^) was not sufficient to ensure normal dendritic length. One should keep in mind that, in the cortex, we find about sevenfold higher protein levels of profilin 2 when compared to profilin 1 (Figure 1A). The loss of all *profilin* alleles produced the most dramatic phenotype, with the shortest dendrites (n-dko). The number of higher-order branches (Figure 7E–G) was more sensitive to profilin levels than the length, with a tendential loss already detectable in mice with a single *profilin 2* allele and a complete loss of quaternary-order branches in the absence of *profilin 2*, independently from *profilin 1* haploinsufficiency or complete loss. On the basal side, the results were quite similar, indicating a robust phenotype affecting the structural cytoskeleton of the neurons. Both the number and the length of the primary dendrites and of the higher-order branches were reduced (Figure 7H–L), again in strict dependence on the expression level of profilin, rather than the specific isoform. In fact, mice with a single *profilin 2* allele had the mildest loss, while mice with a single *profilin 1* allele had a more pronounced phenotype, and mice without any profilin in the neurons had the most severe defect, with complete loss of tertiary-order branches.

In the hippocampus, an accurate quantification of dendritic arborizations through Golgi staining is difficult to achieve due to the higher density of stained neurons and the overwhelming complexity of the dendritic arbors. Yet, overview images of Golgi-stained hippocampal neurons illustrate a similar phenotype as in the cortex (Figure S5), with a progressive loss of complexity as the profilin dosage decreased from the n-*Pfn1*^ko^;*Pfn2*^het^ mice bearing a single *profilin 2* allele to the n-*Pfn1*^het^;*Pfn2*^ko^ mice expressing a single *profilin 1* allele and finally the n-dko, which appeared to have lost all secondary branches.

## 4. Discussion

The *profilin* gene found already in yeast (PFY1) and up to the fly (*D. melanogaster, chickadee*) has undergone several DNA- and RNA-based diversification events during the evolution of the Chordata, and, already in fish (*D. rerio*), at least three isoforms have been found. In mammals, until now, four isoforms have been identified, while, in plants, there are even more. Most eukaryotic cells, therefore, simultaneously express at least two profilins; in particular, neuronal cells in mouse have been shown to express both *profilin 1* and *profilin 2* [7,13]. The basic properties of profilin isoforms appear to be the same: binding of G-actin in a 1:1 complex, catalytically inducing ADP to ATP exchange on the actin monomer, and, in general, positively regulating actin polymerization in concert with other polymerization factors [33]. The difference in their biological function when they are present in the same cell has largely remained elusive. Three aspects should be considered: (1) their absolute expression levels in a given cell; (2) their unique interaction and/or affinity with ligands involved in specific cellular processes [6,34]; (3) specific post-translational regulatory mechanisms (e.g., phosphorylation) that can regulate the different isoforms in response to stimuli [35,36,37,38,39]. In this work, we attempted to address the specific and redundant functions of profilin 1 and 2 in the nervous system of the mouse in two different in vivo settings: (i) cell expansion and differentiation at the beginning of mouse brain development, and (ii) morphology of postmitotic neurons in the mouse adult brain. For this purpose, we employed two separate time- and cell-specific conditional knockout approaches for *profilin 1* in conjunction with a *profilin 2* conventional knockout background.

Our study in the embryonic system supports a mostly redundant function of profilin 1 and 2 in the cell division of neural progenitor cells. Broadly speaking, it is the absolute level of profilins which is critical at this early stage. In fact, we show that, in E13.5 telencephalon, the earliest timepoint where we addressed profilin expression specifically in the neural tissue, the absolute amount of profilin 2 appears to be only about half that of profilin 1, remaining constant until birth. In previous studies, this novel finding was masked by the use of whole embryo or whole head extracts [9,23]. The double *profilin* knockout in the neuroepithelial stem cells (obtained using the *Nes*-Cre driver line) completely arrests telencephalic development between E11.5 and E14.5 and significantly affects mid-/hindbrain development. Corticogenesis in mouse embryos occurs more or less between E11 and E18 and is regulated by the cycles of cell division of the neural precursor cells (also known as radial glia) that can divide both symmetrically to maintain their pool and asymmetrically to implement neurogenesis, as well as astrogenesis [40]. We show that, in our mouse model, profilin 1 protein depletion from the neuroepithelial cells is seemingly complete at E11.5, therefore it is not surprising that the expansion of the neuroepithelial stem-cell pool, occurring before E11, is not significantly affected in *profilin* double knockout embryos or *Pfn2* haploinsufficient embryos, which look quite normal following histological analysis at E11.5. The cell division arrest, therefore, starts in apical radial glia cells in this mouse model, in all three brain vesicles, affecting both the maintenance of the NPCs pool and the generation of the majority of the neurons. This results at E14.5–16.5 in the complete absence of the cortex, where proliferation is known to be most important [41], and in the reduced size of the mid-/hindbrain region. Brain size depends on the NPC cell division rate, with increased proliferation of neural stem cells resulting in an enlarged brain [42], and defective proliferation resulting in microcephaly [43,44].

While a single *profilin 1* allele is able to rescue the *profilin* double knockout phenotype and allow viable mice at birth, a single *profilin 2* allele only succeeds in a very partial rescue that does not abolish the embryonic lethal phenotype. Previous work has shown that two *profilin 2* alleles in a *profilin 1*;*Nes*-Cre knockout mouse model [15,18] are sufficient to substantially rescue the cell division phenotype and obtain viable mice, which is in agreement with a redundant role of profilin 1 and 2 in cell division and an allele requirement based on their individual expression level at this developmental stage. Nevertheless, cell adhesion and neuronal migration deficits in the *profilin 1*;*Nes*-Cre mouse model were not sufficiently compensated for by profilin 2 [16], suggesting a more specific role of profilin 1 in migration- and adhesion-dependent cell functions.

The role of profilin in cell division was first uncovered in fission yeast (*S. pombe*) [45], but the strongest evidence has come from the *profilin 1* knockout mouse model [9], where embryonic development was largely arrested between the two- and four-cell stage, a developmental stage where *profilin 2* is not yet expressed. In the present work, we show that the arrest of cell division in NPCs depleted of both profilins occurs in G2 or at the G2/M transition point of mitosis, as suggested by the accumulation of several markers that should be catabolized in M-phase, such as CDK1 tyrosine 15 phosphorylation (inhibitory for cell division progression) and PTTG1 (that blocks chromatid segregation), and supported by the doubling of p-H3-labeled neural precursor cells and the 50% decrease in dividing NPCs with segregated chromatids. Therefore, it is plausible to conclude that profilin-dependent actin dynamics are required in NPCs for the progression of cell division into the M-phase and finally cytokinesis. A minimal required amount of profilin can be provided by a single *profilin 1* allele or by two *profilin 2* alleles, due to their differential embryonic expression levels. Nevertheless, in the presence of both *profilin 2* alleles, in the conditional *profilin 1* knockout model with the *Nes*-Cre driver, the precise regulation of the orientation of the cleavage plane of apical radial glia cells was disrupted [18], showing fine differences even within the basic common functions of profilin 1 and 2 in cell division.

The fact that the different profilin isoforms can compensate for each other in basic cellular functions is not totally surprising. Profilin is present in multiple isoforms already in early evolutionary organisms, such as *D. discoideum*, and the sequence homology between the many different profilin paralogs and orthologs is never particularly high; for example, mouse profilins 1 and 2 share only a 63% identity at the amino-acid level (NCBI blastp), while the identity between chickadee (*D. melanogaster*) and mouse profilin 1 is as low as 29% (NCBI blastp). What typically characterizes all profilins, nevertheless, is their secondary and tertiary structure (for a review, see [46]). All the crystal structures determined until now are highly superimposable. This is due to the fact that all profilins possess a seven-strand flat beta sheet core, on one side of which, together with two alpha helices, the actin-binding domain is formed, while, on the opposite side, the N- and C-terminal helices come close together to form the SH3-like poly-l-proline-binding domain. Lastly, all profilin ligand interactions are negatively regulated by the binding of phosphatidylinositol phosphates. The structural identity of the profilin orthologs from yeast to man has preserved profilin’s fundamental role in actin dynamics for basic functions such as cell division, cell adhesion, and cell migration to the point that null phenotypes can be rescued by evolutionarily very distant profilins [47]. It is, therefore, not unreasonable to also expect partial functional overlap among paralogs. Of course, the amino-acid sequence diversity in the paralogs can introduce fine functional differences, such as what has been observed between profilin 1 and 2 in the orientation of the radial glia cleavage plane discussed above, or add specific functions.

Why the complete loss of profilin, accompanied by a consistent loss of actin and a general reduction in actin dynamics, results in a cell-cycle arrest in the G2-phase or G2/M transition, could have at least two explanations. First, the genetic ablation of both *profilin* genes in neuroepithelial cells might activate a mitotic actin checkpoint. There has been some evidence for such a mechanism, first described 20 years ago in fission yeast [48], and later shown in different cell culture systems [49,50], using actin-depolymerizing drugs. Only in one report has the mechanism been proposed in vivo, in *filamin A* knockout mice [43], which nevertheless displayed only a mild microcephaly phenotype due to a delay of cell division. The double *profilin* knockout mouse model shows a severe mitosis arrest, with lack of disinhibition of the CDK1 kinase by Tyr15 dephosphorylation, a common signature found in all the abovementioned systems where actin dynamics were disrupted. A second explanation for the severe mitotic arrest of NPCs in double *profilin* knockout embryos is that a minimum profilin level is required for interkinetic nuclear migration (INM), a characteristic process occurring in many neuroepithelial and epithelial thin proliferating layers to increase the cell density and the efficiency of cell division [41]. In radial glia cells, mitosis can occur only at the apical surface, close to the ventricle, since the centrosome, necessary for spindle formation, is located apically in these cells to support cilia extension into the ventricle. As soon as cell division is complete, the nucleus is translocated toward the basal side, creating a pseudostratification of the cells that increases compactness and leaves space at the apical surface for other cells to continue dividing [41]. Furthermore, the S-phase occurs at the basal side, but then the nucleus before nuclear membrane breakdown must be moved to the apical surface. Translocation of the nucleus from the basal to the apical region in NPCs must occur quickly and has been shown to depend on microtubules, as well as actin dynamics [44,51,52,53,54]. It is, therefore, plausible that, in the absence of profilins, this translocation cannot occur or is severely slowed down, physically impeding cell division. A similar phenotype was observed in conditional *cofilin 1* knockout embryos [44], albeit with a less severe phenotype due to partial functional compensation by the *cofilin 1* paralog, actin-depolymerizing factor (*Adf*). The apparent thickening of the p-H3-stained layer in *profilin* double knockout E11.5 embryos supports this view. The ectopic p-H3 staining in the basal region of the cortical primordium is also in agreement with this explanation. It indicates a defoliation process of the ventricular zone cell layer due to crowding-induced mechanical stress [55,56], which can be caused by impaired INM, improper pseudostratification of the cells, and increased number of cells in G2 phase, when they occupy the highest volume. This defoliation process results in the complete loss of dividing cells in the VZ and eventually the absence of the cortex in E16.5 *profilin* double knockout embryos.

Changing perspective from embryonic development to adult brain morphology, another novel and fascinating aspect of versatile profilin function emerged. In early development and cell-cycle control, profilin 1 is the driving isoform in terms of expression and activity, with profilin 2 behaving more as a bystander that can support the same functions if expressed at a sufficient level. After birth, in mature postmitotic neurons, the two isoforms profilin 1 and profilin 2 exchange roles (or importance), with profilin 2 now becoming the limiting factor in the context of cell morphology and function and profilin 1 retaining a partially compensating activity due to a much lower expression level. Structural defects occur in the *profilin* knockout neurons at 3 months of age, such as shortening of dendritic branches and loss of dendritic arborization complexity, with a profilin dosage-dependent severity, while no specific defects seem to occur depending on the deleted *profilin* isoform. Therefore, profilin in postmitotic neurons appears to be necessary for the cytoskeletal support of the complex neuronal structure. In adult mice, profilin 2 levels are markedly higher than profilin 1 levels [6]; in particular, in the cortex, profilin 2 appears to be about sevenfold higher, in good agreement with the previous data. Accordingly, the neuronal phenotype is less severe when a single *profilin 2* allele that can express more profilin is present, more severe when a single *profilin 1* allele, able to express much less profilin, is still present, and finally the most severe when both profilins are depleted. The phenotype dependence on the specific profilin isoform is the opposite of that in embryonic stages, but conceptually identical, due to the reversed quantity ratio. A function of profilin 2 in supporting neuronal morphology has been previously shown using a knockdown approach in hippocampal cultured neurons [57], but the redundant function of profilin 1 in supporting dendritic complexity was not recognized. One possibility could be an insufficient ectopic overexpression of profilin 1, difficult to evaluate in single cells; alternatively, it is possible that, in isolated cultured neurons, profilin isoforms are not equivalent. In our model, the collapse of the dendritic arbor in the absence of both profilins appears to be due to the considerable loss of actin dynamics, as reflected by the large reduction in actin levels and the alteration of F/G-actin ratios. Considering that neurons in the cortex represent only 25–30% of the cells [31], the 40% actin reduction calculated on a total cortical extract suggests a significant loss of actin in *profilin* double knockout neurons. In addition to the loss of total actin, the filamentous to monomeric actin ratio was decreased, accounting for a substantial loss of structural F-actin in *profilin* double knockout neurons. The model shows the importance and the necessity for structural actin in the arborization of postmitotic neurons, in addition to the well-recognized role of actin dynamics in synaptic plasticity. In the Golgi-stained images, it is possible to observe that, in the genotypes lacking *profilin 2*, the density of the stained neurons is decreased. Neuronal death following collapse of the dendritic arborization is plausible and could proceed according to mechanisms similar to transneuronal degeneration [58], since the collapsing dendritic arbors would lose connectivity, and it is a general principle that nerve cells need to be integrated into a functional network and receive trophic signals from other neurons in order to remain viable.

In conclusion, we show here that basic cellular functions in mouse NPCs and neurons can be supported by both profilin 1 and 2, and the main requirement is a threshold profilin level. By no means does the work presented here exclude specific functions in which one profilin isoform is favored or uniquely required. Here, we simply provide two aspects of profilin function—cell proliferation and pyramidal neuron morphogenesis—where the two profilin isoforms can, in principle, compensate for each other. There have already been described activities which suggest additional and rather specific functions of profilins. One example might be cell adhesion and neuronal migration that appear to selectively require profilin 1 [15,16]. Similarly, synaptic transmission appears to mainly depend on profilin 2, according to the hyperexcitability phenotype of the *profilin 2* knockout mouse model and the specific interaction of profilin 2 with synaptic proteins [6,7,59]. We would like to predict that the ligand interaction network and regulatory post-translational mechanisms of the different profilin isoforms will shed more light on the very (cell) specific aspects of profilin isoform function.

## Supplementary Materials

The following are available online at https://www.mdpi.com/article/10.3390/cells10092310/s1. Figure S1. Mice mating schemes with the indication of the obtained genotypes and genotyping strategy; Figure S2. Profilin 1 and profilin 2 levels are strongly decreased in E11.5 mutant embryos and affect actin and Ser3 phosphorylation of cofilin 1; Figure S3. Mitotic neural precursor cells (NPCs) in the midbrain VZ are disarrayed and increased in E11.5 n-dko embryos; Figure S4. Profilin 1 and 2 expression in the cortex of P80–90 profilin mutants; Figure S5. Golgi staining of hippocampus shows a similar phenotype to the cortex.
