# Supplementary material for: Specificity and Redundancy of Profilin 1 and 2 Function in Brain Development and Neuronal Structure
Source: Cells. 2021 Sep 3;10(9):2310. doi: 10.3390/cells10092310 (PMC8467068; doi:10.3390/cells10092310)
Supplement: Supplementary file 1 [file cells-10-02310-s001.zip › cells-1344119-supplementary.pdf]

Supplementary Figures.

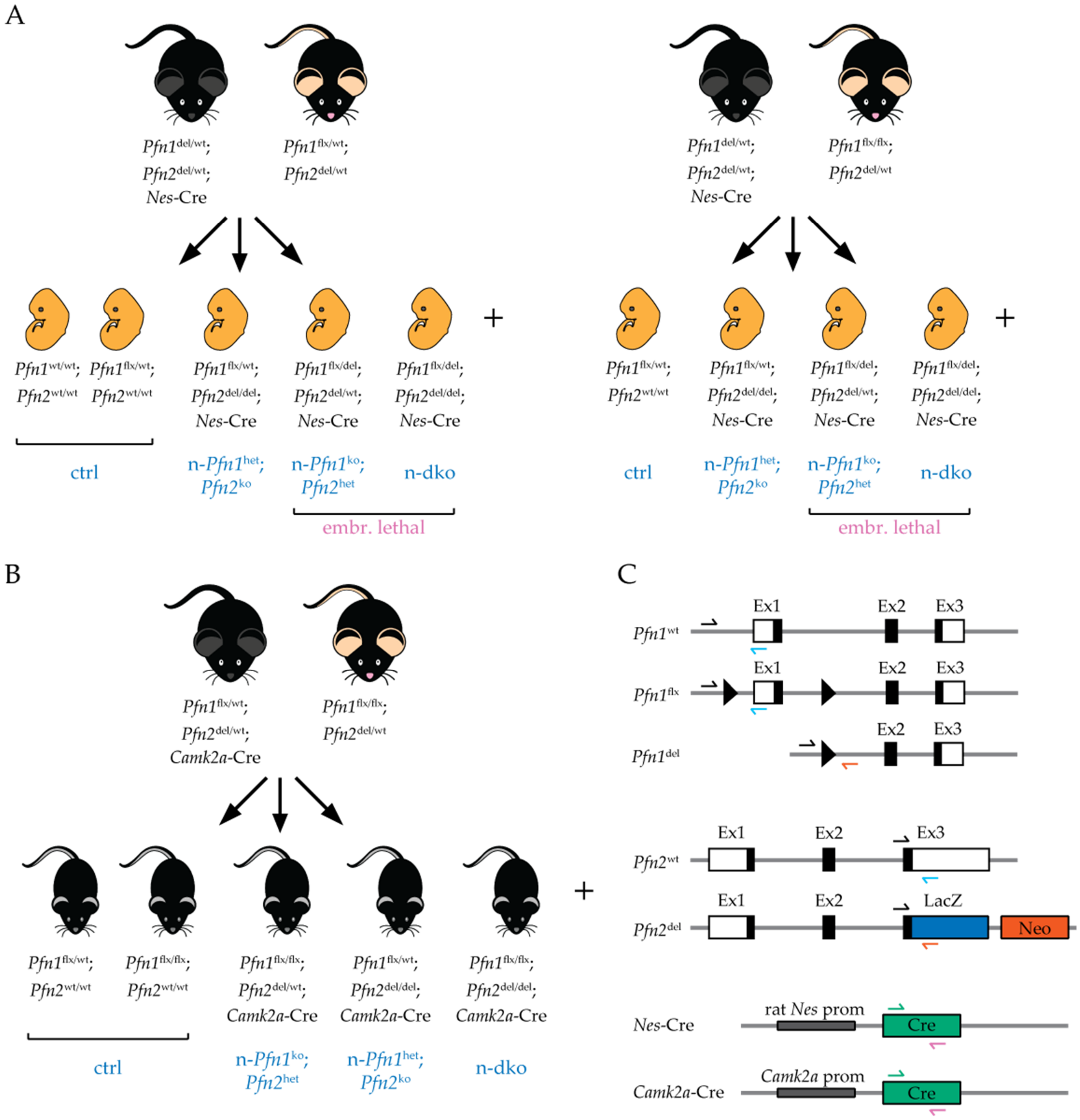

Figure S1. Mice mating schemes with the indication of the obtained genotypes and genotyping strategy. (A) Mating scheme and genotypes obtained for the embryonic studies. Note that the *Nes*-Cre allele is only combined to the *Pfn1*^{ko} allele in breeding pairs since we observed germline deletion of the *Pfn1*^{flx} allele with this Cre line. In blue are indicated the genotypes short names used conventionally in this work. (B) Mating scheme and genotypes obtained for the adult neuronal studies. As in (A) in blue are indicated the genotypes short names. Both Cre lines are transgenic insertions and have been always used

in heterozygosity, therefore the presence of the Cre allele is indicated by writing the line name in the genotype. The + in (A) and (B) next to the genotypes used in this work indicates the existence of additional genotypes arising from the same breeding pair, which were not useful for the present work. (C) Schematic representation of the *Pfn1*, *Pfn2*, *Nes*-Cre and *Camk2a*-Cre alleles and their genotyping strategy. Within each gene wt and flox alleles are recognized by the black-blue primers, deleted alleles by the black-orange pairs and both Cre alleles by the same green-pink primer pair on the Cre sequence.

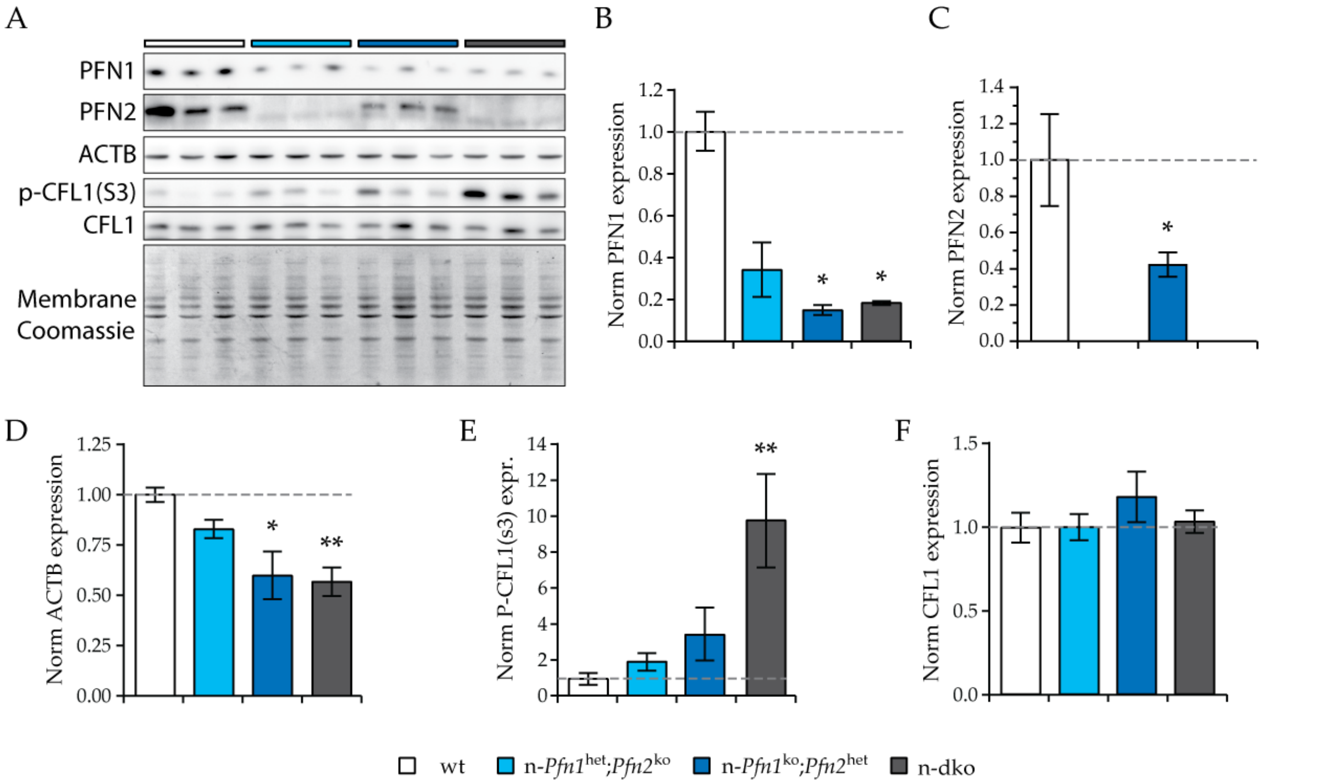

Figure S2. Profilin 1 and profilin 2 levels are strongly decreased in E11.5 mutant embryos and affect actin and Ser3 phosphorylation of cofilin 1. (A) Western blots on E11.5 embryo head total extracts for the indicated proteins. Genotypes of the extracts are indicated by color-coded bars, as in the legend. (B) Normalized quantification histogram of profilin 1 levels in the mutant embryos. A remarkable loss of profilin 1 occurs already in heterozygote embryos (n-*Pfn1*^{het};*Pfn2*^{ko}) and in the n-*Pfn1*^{ko};*Pfn2*^{het} and n-dko embryos only 15-20 % of profilin 1 is left. (C) Normalized quantification histogram of profilin 2 levels in the mutant embryos. (D) Normalized quantification histogram of β-actin (ACTB) levels in the mutant embryos shows a significant loss of actin in proportion to profilin 1 loss. At this developmental stage there is still very little profilin 2. (E,F) Normalized quantification histograms of Ser3-phosphorylated cofilin 1 (p-CFL1(S3)) and cofilin 1 (CFL1) levels, respectively, in the mutant embryos. A strong inactivation of cofilin 1 occurs in n-dko embryos. For each protein, one-way ANOVA followed by Dunnett's post-hoc test to compare mutants to ctrl was applied; n = 3 for each genotype. * $p \leq 0.05$, ** $p \leq 0.01$.

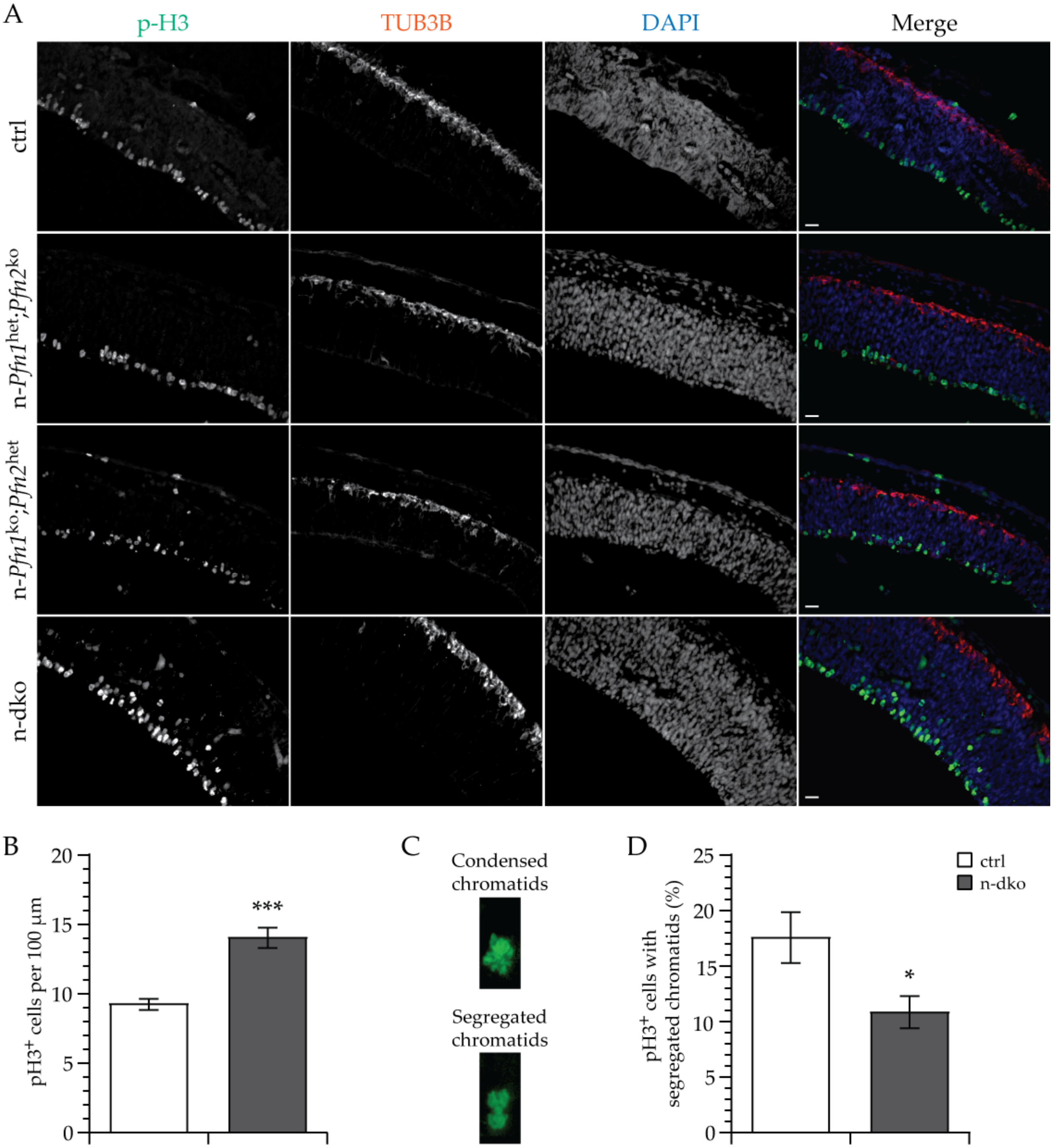

Figure S3. Mitotic neural precursor cells (NPCs) in the midbrain VZ are disarrayed and increased in E11.5 n-dko embryos. (A) Immunofluorescence stainings of E11.5 cryosections with anti-p-H3 (phospho-histone 3) antibodies, green, to label dividing NPCs, anti-TUB3B (β3-tubulin) antibody, red, to label differentiated neurons on the pial surface, and DAPI, blue, to label cell nuclei, show a disarrayed layer of dividing NPCs and ectopic p-H3 labelling in the midbrain region of n-dko as well as n-*Pfn1*^{ko};*Pfn2*^{het} embryos. On the other hand, embryos carrying a single *Pfn1* allele, n-*Pfn1*^{het};*Pfn2*^{ko}, appear unaffected. (B) Quantification of p-H3 positive cells in the VZ of E11.5 embryos shows increased

linear density in n-dko profilin mutants (14.05 ± 0.73 / 100 µm) compared to controls (9.50 ± 0.40 / 100 µm, two-sided Welch's t-test $p < 0.001$); n = 8/2 sections/embryos for ctrl and n = 11/2 for n-dko. (C) Examples of p-H3-labelled condensed and segregated chromatids in NPCs, the latter indicating the progression of cell division to the telophase stage. (D) Quantification of p-H3+ NPCs with segregated chromatids shows a significant 40 % decrease in n-dko embryos (10.81 % ± 1.45 % compared to 17.52 % ± 2.29 % in controls, two-sided Welch's t-test, p=0.0215); n = 8/2 sections/embryos for ctrl and n=11/2 for n-dko. * $p \leq 0.05$, *** $p \leq 0.001$. Scale bar 20 µm.

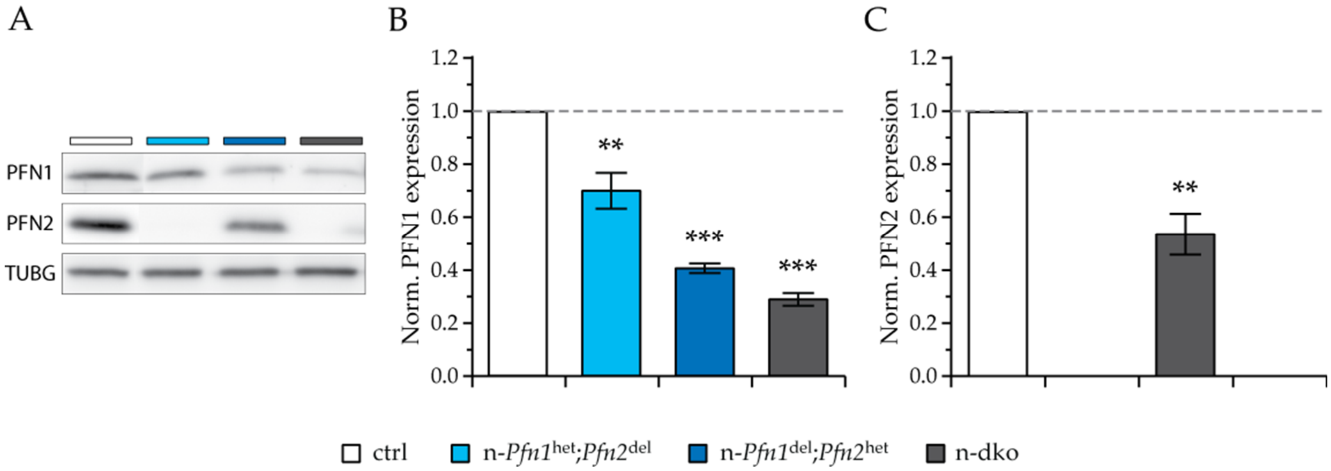

Figure S4. Profilin 1 and 2 expression in the cortex of P80-90 profilin mutants. (A) Representative western blots of profilins in the 4 genotypes indicated by color-coded bars, as in the legend. For calibration of the samples γ-tubulin (TUBG) was used. (B) Normalized quantification histogram of profilin 1 levels in the cortices of the mutant mice. 30 % of profilin 1 is lost in n-*Pfn1*^{het};*Pfn2*^{ko} mice, heterozygote for *profilin 1* (p = 0.0013), while 60 % is lost in cortices of the single *profilin 1* allele mutant n-*Pfn1*^{ko};*Pfn2*^{het} ($p < 0.0001$) and an additional 10 % is lost in n-dko animals ($p < 0.0001$, one-way ANOVA with Dunnett's post-hoc test to compare mutants to ctrl). (C) Normalized quantification histogram of profilin 2 levels in the cortices of the mutant mice (p = 0.002, one-sided Welch's t-test,); n = 3 for each genotype. ** $p \leq 0.01$, *** $p \leq 0.001$.

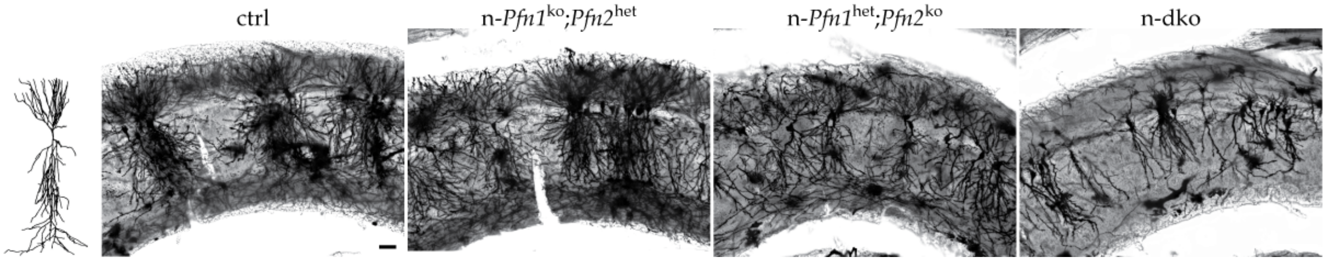

Figure S5. Golgi staining of hippocampus shows a similar phenotype to the cortex. Sample overview images of Golgi-stained hippocampi from P80-90 mice show loss of dendritic arborization in line with the loss of profilin. n-*Pfn1*^{ko};*Pfn2*^{het} mice appear virtually normal, but n-*Pfn1*^{het};*Pfn2*^{ko} mice with only one *profilin 1* allele, expressing 7 times less profilin than the previous genotype, appear strongly affected. N-dko mice, lacking all profilin in the pyramidal neurons, have the most severe phenotype. On the left side, schematic representation of a reconstructed Golgi-stained CA1 pyramidal neuron. Scale bar 40 µm.
